# Supplementary material for: Choosing fit-for-purpose biodiversity impact indicators for agriculture in the Brazilian Cerrado ecoregion
Source: Nat Commun. 2025 Feb 20;16:1799. doi: 10.1038/s41467-025-57037-9 (PMC11842713; doi:10.1038/s41467-025-57037-9)
Supplement: Supplementary file 1 — Supplementary Information [file 41467_2025_57037_MOESM1_ESM.pdf]

Choosing fit-for-purpose biodiversity impact indicators for agriculture in the Brazilian Cerrado ecoregion

Gabriela Rabeschini, U. Martin Persson, Chris West, Thomas Kastner

Supplementary Tables

Supplementary Table 1. Description of the land use and land cover (LULC) classes used in this study from Mapbiomas maps Collection 7.0

Category	Mapbiomas LULC class	Mapbiomas class description
Natural land cover		
	Forest	Types of vegetation with a predominance of tree species, with continuous canopy formation (Riparian Forest, Gallery Forest, Dry Forest and Cerradão), in addition to semi-deciduous seasonal forests.
	Savanna	Savanna formations with defined arboreal and shrub-herbaceous strata (Cerrado in the strict sense: dense Cerrado, typical Cerrado, sparse Cerrado and rocky Cerrado).
	Mangrove	Dense, evergreen forest formations, frequently flooded by the tide and associated with the coastal Mangrove ecosystem.
	Wetland	Vegetation with a predominance of herbaceous stratum subject to seasonal flooding (*e.g. Campo Úmido*) or under fluvial/lacustrine influence (*e.g. Brejo*). In some regions, the herbaceous matrix occurs associated with tree species of savanna formation (*e.g. Parque de Cerrado*) or palm trees.
	Grassland	Grassland formations with a predominance of herbaceous stratum (*campo sujo*, *campo limpo* e *campo rupestre*) and some areas of savanna formations such as the rocky Cerrado.
	Rocky outcrop	Rocks naturally exposed on the earth's surface without soil cover, often with partial presence of rocky vegetation and steep slopes.
	Beach, dune and salt flat	Sandy strips, bright white in color, where there is no predominance of vegetation of any type.
Anthropogenic land use		

Agriculture	Pasture	Pasture area, predominantly planted, linked to agricultural activity. Natural pasture areas are predominantly classified as grassland formations that may or may not be grazed
	Soy	Areas cultivated with soybean crops
	Sugarcane	Areas cultivated with sugarcane crops
	Rice	Areas cultivated with rice crops
	Cotton	Areas cultivated with cotton crops
	Other temporary crops	Areas occupied by short or medium-term agricultural crops, generally with a vegetative cycle of less than one year, which after harvest require new planting to produce
	Coffee	Areas cultivated with coffee crops
	Citrus	Areas cultivated with citrus crops
	Other perennial crops	Areas occupied by agricultural crops with a long vegetative cycle (more than one year), which allow successive harvests without the need for new planting
	Tree plantation	Arboreal species planted for commercial purposes (e.g. pine, eucalyptus, araucaria)
	Mosaic of uses	Areas of agricultural use where it was not possible to distinguish between pasture and agriculture.
Urban		Areas with significant density of buildings and roads, including areas free of buildings and infrastructure.
Other	Mining	Areas related to industrial or artisanal mineral extraction (mining), with clear exposure of the soil due to human activity.
	Other non-vegetated areas	Areas of non-permeable surfaces (infrastructure, urban expansion or mining) not mapped in their classes and regions of exposed soil in natural areas or in areas of cultivation in the off-season.

Supplementary Table 2. Expected percentage population decline over 10 years or three generations from combinations of scope and severity scores per threat. For further details, see ref.[1].

Severity							
		Very rapid declines	Rapid declines	Slow, significant declines	Negligible declines	No decline	Causing/ could cause fluctuations

Scope	Whole (>90%)	62	24	10	1	0	10
	Majority (50-90%)	52	18	9	0	0	9
	Minority (<50%)	24	7	5	0	0	5

Supplementary Figures

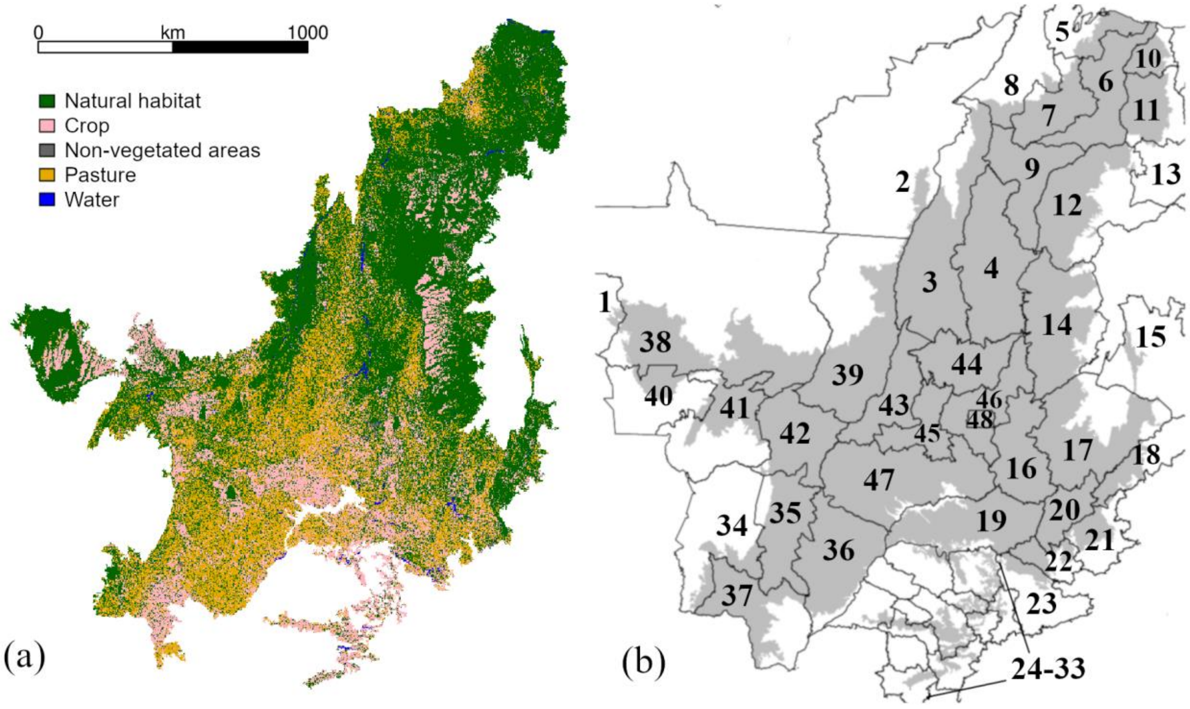

Supplementary Figure 1. **Land use and land cover and mesoregions of the Cerrado.** (a) Distribution of the land use and land cover found in the Cerrado region by 2021. “Natural habitat” includes forest formation, savanna formation, mangrove, wetland, grassland, beach and dune, rocky outcrop and salt flat, and covered around 52% of the region. “Crop” includes forest plantation, sugar cane, mosaic of agriculture and pasture, soybeans, rice, coffee, citrus, cotton, other temporary crops and other perennial crops, and covered around 20% of the region. “Non-vegetated area” includes urban infrastructure, mining and other non-vegetated areas, and covered 0.96% of the region. Pasture covered around 26% of the region. “Water” includes river, lake and

ocean and aquaculture, and covered 0.73% of the region. Categories follow the land use and land cover classification of Mapbiomas platform (Collection 7.0). (b) Mesoregions found across the Cerrado territorial extension: 1 - Leste Rondoniense; 2 - Sudeste Paraense; 3 - Ocidental do Tocantins; 4 - Oriental do Tocantins; 5 - Norte Maranhense; 6 - Oeste Maranhense; 7 - Centro Maranhense; 8 - Leste Maranhense; 9 - Sul Maranhense; 10 - Norte Piauiense; 11- Centro-Norte Piauiense; 12 - Sudoeste Piauiense; 13 - Sudeste Piauiense; 14 - Extremo Oeste Baiano; 15 - Centro Sul Baiano; 16 - Noroeste de Minas; 17 - Norte de Minas; 18 - Jequitinhonha; 19 - Triângulo Mineiro/Alto Paranaíba; 20 - Central Mineira; 21 - Metropolitana de Belo Horizonte; 22 - Oeste de Minas; 23 - Sul/Sudoeste de Minas; 24 - São José do Rio Preto; 25 - Ribeirão Preto; 26 - Bauru; 27 - Araraquara; 28 - Piracicaba; 29 - Campinas; 30 - Presidente Prudente; 31 - Marília; 32 - Assis; 33 - Itapetininga; 34 - Pantanais Sul Mato-grossense; 35 - Centro Norte de Mato Grosso do Sul; 36 - Leste de Mato Grosso do Sul; 37 - Sudoeste de Mato Grosso do Sul; 38 - Norte Mato-grossense; 39 - Nordeste Mato-grossense; 40 - Sudoeste Mato-grossense; 41 - Centro-Sul Mato-grossense; 42 - Sudeste Mato-grossense; 43 - Noroeste Goiano; 44 - Norte Goiano; 45 - Centro Goiano; 46 - Leste Goiano; 47 - Sul Goiano; 48 - Distrito Federal.

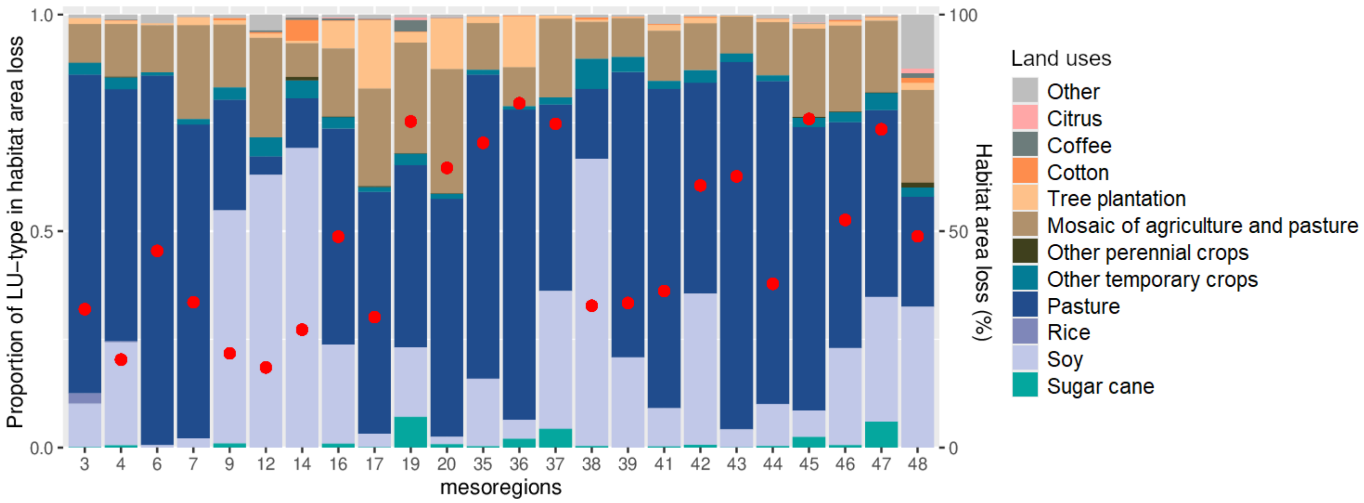

Supplementary Figure 2. **Attribution of habitat area loss to specific land uses.** Average percentage of habitat area size loss (red dots) and share of the land use (LU) types in the total loss in habitat area size for vertebrate species in geographical mesoregions within the Cerrado by 2021. The proportion of loss in habitat area size due to specific agricultural LU was estimated for species

that cannot live in agricultural LUs and that lost habitat area size. For each pixel where the species lost habitat, the total change in area for all the LULC types present in the pixel was calculated and the gain in area of each non-suitable LULC type was estimated as a proportion of the total gain in area for all non-suitable LULC types. The contribution of each LULC type to the loss in habitat area was then assumed to be equivalent to the proportion of the gain in area for this LULC type within the pixel. For instance, suppose a 25 km^2 pixel is inhabited by a species that can only live in wetland and savanna. In this pixel, arable land area increased 3 km^2 and urban area increased 2 km^2 by 2021. Arable land would then be assumed to have contributed to 60% of this species' loss in suitable habitat in that pixel (3 / (3+2) = 0.6). Mesoregions (see Supplementary Fig. 1 for locations): 3 - Ocidental do Tocantins; 4 - Oriental do Tocantins; 6 - Oeste Maranhense; 7 - Centro Maranhense; 9 - Sul Maranhense; 12 - Sudoeste Piauiense; 14 - Extremo Oeste Baiano; 16 - Noroeste de Minas; 17 - Norte de Minas; 19 - Triângulo Mineiro/Alto Paranaíba; 20 - Central Mineira; 35 - Centro Norte de Mato Grosso do Sul; 36 - Leste de Mato Grosso do Sul; 37 - Sudoeste de Mato Grosso do Sul; 38 - Norte Mato-grossense; 39 - Nordeste Mato-grossense; 41 - Centro-Sul Mato-grossense; 42 - Sudeste Mato-grossense; 43 - Noroeste Goiano; 44 - Norte Goiano; 45 - Centro Goiano; 46 - Leste Goiano; 47 - Sul Goiano; 48 - Distrito Federal. Source Data is provided in ref[2].

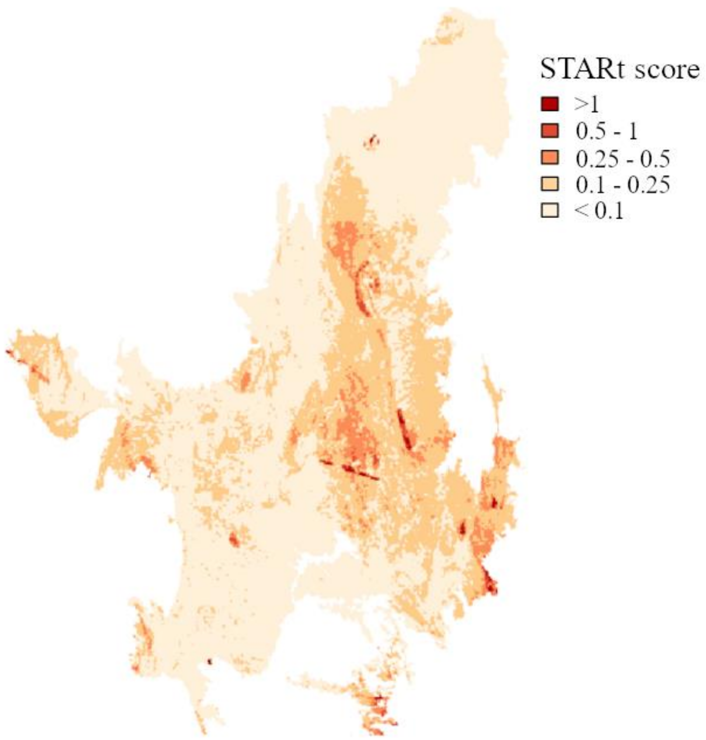

Supplementary Figure 3. **Threat abatement score without weighting.** Global STAR threat abatement ($STAR_T$) score calculated without the weighting factor for extinction risk classification (W_s in eq. 8), disaggregated at 5-km pixel resolution. Higher scores are given to locations with greater shares of the species' remaining habitat area and, in the case of this study, with more species for which the relative contribution of agricultural threats among the other threats is greater. Source Data is provided in ref[2].

Supplementary References

1. Mair, L. *et al.* A metric for spatially explicit contributions to science-based species targets. *Nat. Ecol. Evol.* **5**, 836–844 (2021).

2. Rabeschini, G. (2024). Code&Data_'Choosing fit-for-purpose biodiversity impact indicators for agriculture in the Brazilian Cerrado ecoregion' (version_1) [Data set]. Zenodo. https://doi.org/10.5281/zenodo.11352608 (2024).
