# Supplementary material for: Choosing fit-for-purpose biodiversity impact indicators for agriculture in the Brazilian Cerrado ecoregion
Source: Nat Commun. 2025 Feb 20;16:1799. doi: 10.1038/s41467-025-57037-9 (PMC11842713; doi:10.1038/s41467-025-57037-9)
Supplement: Supplementary file 3 — Reporting Summary [file 41467_2025_57037_MOESM3_ESM.pdf]

nature portfolio

Corresponding author(s): Gabriela Rabeschini, Martin Persson

Last updated by author(s): Feb 5, 2025

Reporting Summary

Nature Portfolio wishes to improve the reproducibility of the work that we publish. This form provides structure for consistency and transparency in reporting. For further information on Nature Portfolio policies, see our Editorial Policies and the Editorial Policy Checklist.

Statistics

For all statistical analyses, confirm that the following items are present in the figure legend, table legend, main text, or Methods section.

n/a	Confirmed	
☐	☒	The exact sample size (*n*) for each experimental group/condition, given as a discrete number and unit of measurement
☒	☐	A statement on whether measurements were taken from distinct samples or whether the same sample was measured repeatedly
☒	☐	The statistical test(s) used AND whether they are one- or two-sided<br>*Only common tests should be described solely by name; describe more complex techniques in the Methods section.*
☒	☐	A description of all covariates tested
☒	☐	A description of any assumptions or corrections, such as tests of normality and adjustment for multiple comparisons
☐	☒	A full description of the statistical parameters including central tendency (e.g. means) or other basic estimates (e.g. regression coefficient) AND variation (e.g. standard deviation) or associated estimates of uncertainty (e.g. confidence intervals)
☒	☐	For null hypothesis testing, the test statistic (e.g. *F*, *t*, *r*) with confidence intervals, effect sizes, degrees of freedom and *P* value noted<br>*Give P values as exact values whenever suitable.*
☒	☐	For Bayesian analysis, information on the choice of priors and Markov chain Monte Carlo settings
☒	☐	For hierarchical and complex designs, identification of the appropriate level for tests and full reporting of outcomes
☒	☐	Estimates of effect sizes (e.g. Cohen's *d*, Pearson's *r*), indicating how they were calculated

Our web collection on statistics for biologists contains articles on many of the points above.

Software and code

Policy information about availability of computer code

Data collection	No software / code was used for data collection.
Data analysis	All analyses and maps were done with R Studio software (R Core Team version 4.2.2 2021), with packages terra (version 1.7-71), raster (version 3.6-26), data.table (version 1.15.4), exactextractr (version 0.10.0) and caret (version 6.0-94). Codes are available at Zenodo repository (https://doi.org/10.5281/zenodo.11352608).

For manuscripts utilizing custom algorithms or software that are central to the research but not yet described in published literature, software must be made available to editors and reviewers. We strongly encourage code deposition in a community repository (e.g. GitHub). See the Nature Portfolio guidelines for submitting code & software for further information.

Data

Policy information about availability of data

All manuscripts must include a data availability statement. This statement should provide the following information, where applicable:

- Accession codes, unique identifiers, or web links for publicly available datasets
- A description of any restrictions on data availability
- For clinical datasets or third party data, please ensure that the statement adheres to our policy

All data on species were downloaded from IUCN Red List (version 2022-2) (https://www.iucnredlist.org/).
All data on land cover and land use were downloaded from Mapbiomas Collection 7.0 (https://brasil.mapbiomas.org/downloads/).

Digital elevation models were downloaded from Open DEM (https://www.opendem.info/link_dem.html).
Shapefiles of geographical borders were downloaded from the Brazilian Institute of Geography and Statistics' website (https://www.ibge.gov.br/en/geosciences/territorial-organization/territorial-meshes/2786-np-municipal-mesh/18890-municipal-mesh.html?=&t=acesso-ao-produto).
All data produced in the study as well as Source Data for Table 2, Figures 1, 2, 3, 4 and 5, and Supplementary Figures 2 and 3 are available on Zenodo repository (https://doi.org/10.5281/zenodo.11352608). Source Data for generating Supplementary Figure 1 is publicly available on Mapbiomas platform (https://brasil.mapbiomas.org/en/) and the Brazilian Institute for Geography and Statistics' website (https://www.ibge.gov.br/en/geosciences/territorial-organization/territorial-meshes/).

Research involving human participants, their data, or biological material

Policy information about studies with human participants or human data. See also policy information about sex, gender (identity/presentation), and sexual orientation and race, ethnicity and racism.

Reporting on sex and gender	N/a
Reporting on race, ethnicity, or other socially relevant groupings	N/a
Population characteristics	N/a
Recruitment	N/a
Ethics oversight	N/a

Note that full information on the approval of the study protocol must also be provided in the manuscript.

Field-specific reporting

Please select the one below that is the best fit for your research. If you are not sure, read the appropriate sections before making your selection.

☐ Life sciences ☐ Behavioural & social sciences ☒ Ecological, evolutionary & environmental sciences

For a reference copy of the document with all sections, see nature.com/documents/nr-reporting-summary-flat.pdf

Ecological, evolutionary & environmental sciences study design

All studies must disclose on these points even when the disclosure is negative.

Study description	This study uses standardized input data from publicly available data sources for applying three biodiversity impact indicators—the countryside Species Area Relationship (cSAR) model, the Species Threat Abatement and Restoration (STAR) metric, and the Species Habitat Index (SHI)—to estimate agriculture's impact on the biodiversity of terrestrial vertebrates in the Brazilian Cerrado.<br>To apply the metrics, we produced three 5km-resolution rasters of the Area of Habitat (AOH) of 2,185 native terrestrial vertebrate species found in the Cerrado, for contemporary (2021) and recent (1985) land use patterns, as well as for pristine conditions (i.e. assuming land use absence). We used spatial information on species distribution ranges and habitat preferences from IUCN Red List, land use and land cover maps from Mapbiomas (Collection 7.0) and digital elevation models to produce the AOH rasters. We assessed impact in different levels. To know how agriculture in the Cerrado contributes to the species' global extinction risk, we used the STAR approach and global-weighted applications of cSAR and SHI. To know how agricultural land use in a specific region contributes to the risk of species disappearing from it, we used the cSAR and SHI approaches at two levels: at the whole Cerrado ecoregion and in 48 geopolitical mesoregions within Cerrado. Finally, we used the cSAR to assess biodiversity impact at local scale (i.e. 5km pixel-resolution).
Research sample	All terrestrial vertebrate species inhabiting the Brazilian Cerrado ecoregion with range distribution maps available at IUCN Red List.
Sampling strategy	We used the advanced filter tool in IUCN Red List to filter species living within Brazil. Then we cropped their distribution ranges according to Cerrado's extent to get the final species used in the study. We used information on species' habitat preferences from IUCN Red List and land use and land cover maps from Mapbiomas (Collection 7.0) to produce the rasters of species' AOH and used digital elevation models to refine them according to species' elevational ranges.
Data collection	We downloaded all data on species from IUCN Red List (version 2022-2) website (https://www.iucnredlist.org/).<br>We downloaded all data on land cover and land use from Mapbiomas platform (Collection 7.0) (https://brasil.mapbiomas.org/downloads/).<br>Digital elevation models were downloaded from Open DEM (https://www.opendem.info/link_dem.html) and used to refine the Area of Habitat of species.<br>Shapefiles of geographical borders were downloaded from the Brazilian Institute of Geography and Statistics' website (https://www.ibge.gov.br/en/geosciences/territorial-organization/territorial-meshes/2786-np-municipal-mesh/18890-municipal-mesh.html?=&t=acesso-ao-produto).
Timing and spatial scale	This study covers the spatial extension of the Brazilian Cerrado ecoregion and uses land cover and land use configurations considering contemporary (2021), recent past (1985) and pristine (i.e. assuming land use absence) states.

Data exclusions	We excluded exclusively aquatic species and species marked as 'data deficient' on IUCN Red List.
Reproducibility	All data analysis can be reproduced using the code made available at Zenodo repository and applying the same underlying publicly available data described above.
Randomization	N/a
Blinding	N/a

Did the study involve field work? ☐ Yes ☒ No

Reporting for specific materials, systems and methods

We require information from authors about some types of materials, experimental systems and methods used in many studies. Here, indicate whether each material, system or method listed is relevant to your study. If you are not sure if a list item applies to your research, read the appropriate section before selecting a response.

Materials & experimental systems

n/a	Involved in the study
☒	☐ Antibodies
☒	☐ Eukaryotic cell lines
☒	☐ Palaeontology and archaeology
☒	☐ Animals and other organisms
☒	☐ Clinical data
☒	☐ Dual use research of concern
☒	☐ Plants

Methods

n/a	Involved in the study
☒	☐ ChIP-seq
☒	☐ Flow cytometry
☒	☐ MRI-based neuroimaging

Plants

Seed stocks	N/a
Novel plant genotypes	N/a
Authentication	N/a
